# Collagen fleece-bound fibrin sealant is not associated with an increased risk of thromboembolic events or major bleeding after its use for haemostasis in surgery: a prospective multicentre surveillance study

**DOI:** 10.1186/1754-9493-3-13

**Published:** 2009-06-22

**Authors:** Mathias Birth, Joan Figueras, Stéphane Bernardini, Tine Troen, Klaus Günther, Darius Mirza, Frank Viborg Mortensen

**Affiliations:** 1 Klinik für Allgemein-, Viszeral-, Thorax- und Gefässchirurgie, HANSE-Klinikum Stralsund, Stralsund, Germany; 2 Servicio de Cirugia General, Dr. Josep Trueta Hospital, IDiBGi, Girona, Spain; 3 Service d'Urologie, CHU Saint Jacques, Besançon, France; 4 Biostatistics, Nycomed, Roskilde, Denmark; 5 Abteilung für Allgemein- und Viszeralchirurgie, Klinik Hallerwiese, Nürnberg, Germany; 6 The Liver Unit, Queen Elizabeth Hospital and Birmingham Children's Hospital, Birmingham, UK; 7 Kirurgisk Gastroenterologisk Afdeling L, Aarhus University Hospital, NBG, Aarhus, Denmark

## Abstract

**Background:**

Topical haemostatic agents are used to help achieve haemostasis during surgery when standard surgical techniques are insufficient. The objective of this study was to confirm the safety profile of an equine collagen patch coated with human fibrinogen and human thrombin with particular focus on the occurrence of thromboembolic events (TEEs), major bleeding and immunological events.

**Methods:**

This was a non-interventional, multicentre, prospective, surveillance study in which a collagen fleece-bound fibrin sealant was prescribed in accordance with its marketing authorisation. The decision to use the sealant was based solely on current surgical practice. All patients that received the sealant and provided informed consent were included. TEEs (any coagula-based occlusion in a vessel or the heart identified by symptomatic clinical signs and/or verified by paraclinical examination), major bleeding (any bleeding that required intervention), and immunological events (hypersensitivity including anaphylaxis) that occurred during surgery, post-operative hospital stay or 6 months of follow-up were reported as adverse events. The primary endpoint was the proportion of patients experiencing a confirmed TEE.

**Results:**

A total of 3098 patients were recruited at 227 centres in 12 European countries. The most frequent types of surgery were hepatic (33%), gastrointestinal (16%) and urological (14%) and the main indication for surgery was for primary (35%) or secondary (20%) malignancy. Forty-six patients (1.5%, 95% CI 1.1–2.0%) had at least one TEE during the study. The most commonly reported TEEs were pulmonary embolism or post-procedural pulmonary embolism (n = 18) and deep vein thrombosis (n = 9). There were 64 major bleedings in 62 patients and 9 immunological events in 8 patients.

**Conclusion:**

Collagen fleece-bound fibrin sealant does not appear to be associated with an increased risk of TEEs, major bleeding or immunological events in patients undergoing surgery.

**Trial registration:**

Clinicaltrials.gov number: NCT00285623

## Background

Rapid and effective control of bleeding during surgery reduces blood loss and can help decrease post-operative morbidity and mortality. Ligation, stapling, clipping and electrocautery are all widely used techniques to prevent bleeding. Over the past 20 years, a wide variety of topical haemostatic agents such as fleeces of different origin (collagen-based, e.g. Avitene^®^; gelatine-based, e.g. Surgifoam^®^, Gelfoam^®^; regenerated oxidised cellulose-based, e.g. Surgicel^®^, Curacel^®^), liquid fibrin sealants (e.g. Tisseel^®^, Tissucol^®^, Evicel^®^, Beriplast^®^), albumin and glutaraldehyde bioglue (BioGlue^®^) and synthetic glues (e.g. CoSeal^®^) have increasingly been used in a range of surgical procedures to help achieve haemostasis when standard surgical techniques are insufficient [1-3]. The use of these agents has a beneficial effect on surgical outcomes, including improved haemostasis, fewer complications and reduced duration of post-operative hospital stay [1,2].

TachoSil^® ^is a sterile, absorbable, haemostatic agent that consists of an equine collagen patch coated on one side with human fibrinogen and human thrombin. Unlike other fibrin sealants that require preparation before use, TachoSil is a ready-to-use fixed combination that is activated by moisture on application, providing adherence to the resection surface and haemostasis. The adhesive strength of TachoSil has been shown to be significantly higher than that of liquid fibrin glue [4] and the effect of the fibrinogen and thrombin together with the mechanical support of the collagen patch provides a physiologically extensible and pliable liquid and air tight seal [5]. TachoSil and its predecessor products, TachoComb^® ^and TachoComb H, have been used in a variety of surgical settings since being introduced in the early 1990s. TachoSil is indicated for supportive treatment in surgery for improvement of haemostasis, to promote tissue sealing, and for suture support in vascular surgery where standard techniques are insufficient.

Clinical studies have shown that TachoSil is effective in achieving haemostasis after kidney or liver resection [6,7], as well as preventing air leakage after lung surgery [8,9] and reducing lymphatic fluid production from the mediastinum [10]. TachoSil has also been shown to be safe and well tolerated, with occurrence of adverse events similar in TachoSil and non-TachoSil treated patients in controlled trials [6-10].

The objective of this study was to confirm the established safety profile of TachoSil with particular focus on the occurrence of thromboembolic events (TEEs), major bleeding and immunological events. The possible role of drug interactions in TEEs and major bleeding was also investigated.

## Methods

This international, multicentre, prospective, surveillance study was of a non-interventional design, meaning that TachoSil was prescribed in accordance with the terms of its marketing authorisation. The decision to use TachoSil was made by the surgeon solely on the basis of current clinical practice. No additional diagnostic or monitoring procedures were applied.

Relevant Ethics Committees approved the protocol, and the trial was conducted in accordance with the Declaration of Helsinki, Good Pharmacoepidemiology Practice, the Data Protection Directive and any additional local regulations. A Data Monitoring Committee (DMC), which comprised of three surgeons (KG, DM, FM) not involved in recruiting patients to the study, was established before the protocol was finalised to regularly review the data. All patients provided written informed consent in accordance with local regulations, allowing the collection of personal data, direct data access and data processing. Consent could be provided either before or after surgery but all were obtained before data were entered into the study database. All patients that received TachoSil and provided informed consent were included in this study.

Reportable adverse events (AEs) were any TEE (a coagula-based occlusion in a vessel or the heart identified by symptomatic clinical signs and verified by paraclinical examination, e.g. ultrasound, magnetic resonance or computed tomography scan, or identified by paraclinical examination only [no routine paraclinical examination]), major bleeding (any bleeding that required intervention), or immunological event (hypersensitivity including anaphylaxis) that occurred during surgery, post-operative hospital stay or 6 months of follow-up. Follow-up at 6 months was done either by personal contact (telephone or visit) or by review of patients' medical records by the participating physician. Reportable AEs were coded by system organ class using MedDRA terminology (version 10.1). AE severity was defined as mild (transient symptoms, no interference with daily activities), moderate (marked symptoms, moderate interference with daily activities) or severe (considerable interference with daily activities). Serious AEs were defined as those that resulted in death, were life-threatening, required overnight inpatient hospitalisation or prolongation of existing hospitalisation, resulted in persistent or significant disability/incapacity, involved congenital anomaly or birth defect, or that required intervention to prevent any of the previously listed. Causality between TachoSil and reportable AEs were assessed by the participating physician and defined as probable (good reason and sufficient documentation to assume a causal relationship), possible (causal relationship conceivable), unlikely or not related.

If the reporting physician suspected that any thromboembolic or major bleeding event was possibly or probably caused by a drug interaction between TachoSil and a concomitant medication then this was documented.

Demographic characteristics, known risk factors for TEEs, bleeding and immunological events, ECG data, and surgical indication were documented for all patients. During surgery, the type and volume of blood products used was documented, as was the use of TachoSil. Use of anticoagulant therapy was assessed before, during and after surgery. Concomitant medication use was also recorded before, during and after surgery, as well as during the 6-month follow-up.

### Statistical analysis

The primary endpoint was the proportion of patients (with 95% confidence intervals [CIs]) experiencing a confirmed TEE. All proportions were based on the number of patients exposed at a given timepoint with no adjustment for multiplicity or missing values. The number and percentage of patients experiencing a serious TEE or a TEE at least possibly related to TachoSil were also summarised.

Secondary endpoints included the proportion of patients experiencing an immunological event, the proportion of patients experiencing a major bleeding requiring interventional treatment, and drug interactions with TachoSil resulting in a TEE or major bleeding. Immunological events and major bleedings were summarised in a similar manner to TEEs.

A review of the literature available before the study began suggested that the incidence of TEEs for surgical procedures without the use of TachoSil was 10–15%. On this basis, an observed incidence rate of 10% could be estimated with a precision (2-sided 95% CI) of ± 1.1% and an observed incidence rate of 15% could be estimated with a precision (2-sided 95% CI) of ± 1.3% with a sample size of 3000 patients.

During the study, all written consents were checked and collected data were verified against the source for approximately 10% of patients (patients with an ID number on their case report form with end digit 2, not revealed to sites until after the study) and for all reportable serious AEs.

## Results

### Patient and surgical characteristics

Between June 2005 and June 2007, 3098 patients received TachoSil while undergoing surgery and provided written informed consent to the use of their data. Patient data were collected from 227 surgical departments or clinics in 12 European countries (Germany, 1548 patients; France, n = 461; Spain, n = 343; Greece, n = 161; Austria, n = 146; the Netherlands, n = 119; Sweden, n = 98; Belgium, n = 82; Latvia, n = 76; UK, n = 41; Norway, n = 15; and Denmark, n = 8). Almost all patient consents were obtained after surgery. No patients withdrew their consent during 6 months of follow-up. A total of 179 patients discontinued from the study early (death, n = 156; lost to follow-up, n = 20; other reasons, n = 3).

Over half of the patients were male (56%), most were Caucasian (98%) and their mean ± SD age was 61 ± 14 years. The most frequent types of surgery were hepatic (33%), gastrointestinal (16%) and urological (14%). The main indication for surgery was for primary (35%) or secondary (20%) malignancy. Ten percent of patients received surgery for benign neoplastic disease. Demographic characteristics and surgical variables are summarised in Table 1.

**Table 1:** Baseline demographic and surgical characteristics

	All patients (n = 3098)	Patients with TEE (n = 46)	Patients with major bleeding (n = 62)	Patients with immunological event (n = 8)
Age (years), mean ± SD	60.7 ± 14.0	63.0 ± 10.0	58.6 ± 16.5	59.5 ± 11.8

Gender (female/male), %	43.7/56.3	28.3/71.7	32.3/67.7	37.5/62.5

BMI (kg/m^2^), mean ± SD	26.6 ± 4.9	27.2 ± 4.4	26.3 ± 6.1	26.0 ± 5.4

Type of surgery, n (%):				
Hepatic	1007 (32.5%)	22 (47.8%)	16 (25.8%)	3 (37.5%)
Gastrointestinal	500 (16.1%)	7 (15.2%)	13 (21.0%)	2 (25.0%)
Urological	422 (13.6%)	2 (4.3%)	6 (9.7%)	1 (12.5%)
Vascular	227 (7.3%)	5 (10.9%)	3 (4.8%)	0
Pulmonary	224 (7.2%)	4 (8.7%)	6 (9.7%)	1 (12.5%)
Cardiac	173 (5.6%)	0	5 (8.1%)	1 (12.5%)
Gynaecological	130 (4.2%)	0	1 (1.6%)	0
Neurosurgical	83 (2.7%)	2 (4.3%)	1 (1.6%)	0
Other	424 (13.7%)	4 (8.7%)	10 (16.1%)	0

Indication for surgery, n (%):				
Primary malignancy	1074 (34.7%)	20 (43.5%)	17 (27.4%)	3 (37.5%)
Secondary malignancy	619 (20.0%)	12 (26.1%)	7 (11.3%)	3 (37.5%)
Benign neoplastic disease	306 (9.9%)	2 (4.3%)	7 (11.3%)	0
Other	1098 (35.4%)	12 (26.1%)	31 (50.0%)	2 (25.0%)

Surgery, acute/elective, %	10.2/89.8	10.9/89.1	24.2/75.8	12.5/87.5

Duration of surgery (hours), mean ± SD	3.2 ± 1.8	4.4 ± 2.4	3.7 ± 2.4	4.4 ± 2.8

Patients receiving blood transfusion, n (%)	892 (28.8%)	14 (30.4%)	31 (50.0%)	2 (25.0%)

Number of transfusion units given, mean ± SD	5.2 ± 7.3	9.5 ± 12.1	10.0 ± 15.9	3.5 ± 2.1

Area of TachoSil used (cm^2^), mean ± SD	53.7 ± 40.7	60.1 ± 41.4	51.7 ± 36.3	55.1 ± 40.8

Of 2752 patients who had an ECG before surgery, 17.3% (n = 477, 15.4% of total study population) had abnormal results. Forty-three percent of patients (n = 1327) received anti-coagulant prophylaxis before surgery. Of these, 95% had their coagulation status tested, and of those tested 11% (n = 139) had abnormal coagulation status. During surgery, or between surgery and hospital discharge, 80% of patients (n = 2491) received anti-coagulant prophylaxis, with 20% (n = 440) of the 90% tested having abnormal coagulation status.

A total of 892 patients (29%) received blood transfusion. Erythrocyte concentrate was the most frequently given blood product (n = 736), followed by fresh frozen plasma (n = 362), whole blood (n = 84) and platelets (n = 65). The median volume of blood products given was 3 units (range 1–83 units) (data available for 873 patients). The median area of TachoSil used was 45.6 cm^2 ^(range 0.3–616.3 cm^2^), the size of one standard patch, based on the amount used during surgery without any correction for overlap of multiple patches.

The majority of patients (91%) had at least one baseline risk factor for a TEE (Table 2). A risk factor for bleeding was reported for 8.2% of patients (bleeding or bleeding tendency, 4.6%; abnormal pre-operative coagulation test, 4.5%) and 15.3% of patients had a risk factor for an immunological event (known allergy, 13.4%; any auto-immune disease, 2.6%).

**Table 2:** Patients with known risk factors at baseline for thromboembolic events

Thromboembolic risk factor:	All patients (n = 3098)	Patients with TEE (n = 46)	Patients with no TEE (n = 3052)	P-value*
Any thromboembolic risk factor	2813 (90.8%)	42 (91.3%)	2771 (90.8%)	>0.99

Cardiovascular (CV) risk factor:	2135 (68.9%)	31 (67.4%)	2104 (68.9%)	0.87
Personal/family history of TEEs	64 (2.1%)	2 (4.3%)	62 (2.0%)	0.25
≥ 1 other CV risk factor	2122 (68.5%)	31 (67.4%)	2091 (68.5%)	0.87

Chronic obstructive pulmonary disease	365 (11.8%)	6 (13.0%)	359 (11.8%)	0.82
Cancer	1597 (51.5%)	30 (65.2%)	1567 (51.3%)	0.07

Metabolic/endocrinological risk factor	797 (25.7%)	15 (32.6%)	782 (25.6%)	0.31

Other thromboembolic risk factor	270 (8.7%)	6 (13.0%)	264 (8.7%)	0.29

Abnormal pre-operative ECG	477 (15.4%)	10 (21.7%)	467 (15.3%)	0.22

* From Fishers Exact test of 2 × 2 tables comparing for each risk factor to which extent the risk factor was predictive of the occurrence of TEEs

The most frequently used classes of concomitant medications were heparins (79% of patients), proton pump inhibitors (60%), anilides (e.g. paracetamol) (43%), benzodiazepine derivatives (33%), opioid anaesthetics (32%), cephalosporins and related substances (31%), plain sulphonamides (31%), and other general anaesthetics (28%).

### Safety

A total of 124 AEs were reported; 51 TEEs in 46 patients, 64 major bleedings in 62 patients and 9 immunological events in 8 patients. Thirteen events were considered to be at least possibly related to TachoSil (3 TEEs, 8 major bleedings and 2 immunological events) by study surgeons or other physicians (Table 3).

**Table 3:** Occurrence of thromboembolic, major bleeding and immunological events

	All	Mild	Moderate	Severe	Serious	TachoSil-related*
Thromboembolic events	51	12	19	20	46	3

Major bleeding	64	4	27	33	57	8

Immunological events	9	6	3	0	1	2

*Considered at least possibly-related to TachoSil by the participating physician (probable = good reason and sufficient documentation to assume a causal relationship; possible = causal relationship conceivable). Mild = transient symptoms, no interference with daily activities; moderate = marked symptoms, moderate interference with daily activities; severe = considerable interference with daily activities; serious = resulted in death, life-threatening, required overnight inpatient hospitalisation or prolongation of existing hospitalisation, resulted in persistent or significant disability/incapacity, involved congenital anomaly or birth defect, or that required intervention to prevent any of the previous occurrence of any of the previously listed.

### Thromboembolic events (TEEs)

A total of 46 patients (1.5%, 95% CI 1.1–2.0%) had at least one TEE at any time during the study (primary endpoint).

**Figure 1:**
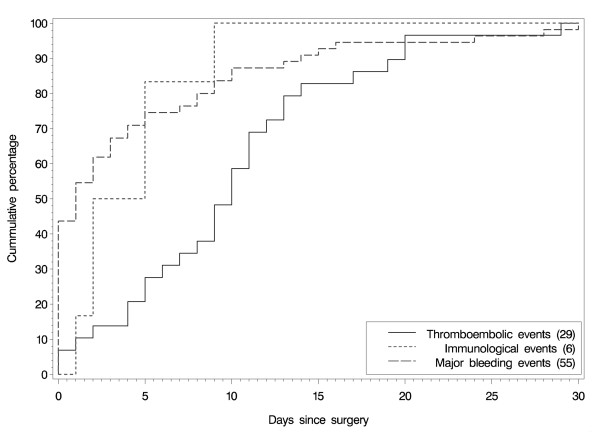
**Occurrence of thromboembolic, major bleeding and immunological events by time from surgery**.

The most commonly reported TEEs were pulmonary embolism or post-procedural pulmonary embolism (18 patients), deep vein thrombosis (n = 9), phlebitis (n = 3), embolism (not otherwise specified) (n = 2), subclavian vein thrombosis (n = 2), portal vein thrombosis (n = 2), thrombosis (not otherwise specified) (n = 2) and myocardial infarction (n = 2). TEEs occurred on the day of surgery in 2 patients, and between the date of surgery and hospital discharge in 23 patients, with the remainder occurring during follow-up. TEEs by time of occurrence (up to one month post-surgery) are shown in Figure 1.

Twelve patients had mild TEEs (12 events), while moderate and severe TEEs each occurred in 17 patients (19 and 20 events, respectively). Forty-one patients had a TEE that was considered serious (46 events).

Three patients had a TEE that was considered at least possibly related to TachoSil by the study surgeon or other physician. Two of these were considered to be serious; one patient had phlebitis in the left leg 11 days after surgery, which was of moderate severity, while another patient had severe, post-procedural pulmonary embolism one week after surgery. Both patients recovered without sequelae. The third TEE considered possibly related to TachoSil was portal vein thrombosis of mild severity with onset two days after surgery. None of the seven TEEs leading to death were considered related to TachoSil.

### Major bleeding

Sixty-two patients (2.0%, 95% CI: 1.5–2.6%) had at least one major bleeding (64 events). Most major bleedings occurred on the day of surgery (n = 24) or between surgery and hospital discharge (n = 28) (Figure 1).

A total of 55 patients had 57 serious major bleeding events, with 8 events in 7 patients considered at least possibly related to TachoSil. These included post-procedural haemorrhage in 3 patients, one on the day of surgery, one the day after surgery and one 4 days after surgery. One post-procedural haemorrhage was severe and 2 were moderate in severity. The other events considered at least possibly related to TachoSil were severe haemothorax, severe haemorrhage, moderately severe splenic haemorrhage, and moderately severe haematoma, all of which occurred on the day of surgery. In addition, drug ineffective was reported as an AE in the patient with moderately severe splenic haemorrhage. All 4 patients recovered, although the patient with haematoma died later from sepsis. None of the 8 major bleeding events leading to death was considered related to TachoSil.

### Immunological events

Eight patients (0.3%) experienced at least one immunological event. The most common immunological events were rash or rash pruritis, which occurred in 3 patients. No immunological events were reported during surgery, while 5 occurred between surgery and discharge. One immunological event was deemed serious but not related to TachoSil (increased white blood cell count). One patient had 2 immunological events considered to be at least possibly related to TachoSil (pyrexia and eosinophilia).

Previous exposure to TachoSil among patients was unknown. Seven patients were reported to be participating or had previously participated in another clinical trial or non-interventional study with TachoSil, while 2 patients received TachoSil on two occasions during this study. None of these patients experienced an immunological event.

### Drug interactions

No drug interactions with TachoSil were suspected by the participating physician as the cause of a TEE or major bleeding. Over 38 000 concomitant medications were recorded, coded and grouped to ATC code level 4, with an average of 12.3 concomitant drugs per patient. To identify possible drug interactions, odds ratios (OR) were calculated post-hoc based on drug classes and number of exposed patients with and without events. With Hochberg correction for multiple testing, the only significant OR was for natural opium alkaloids and TEEs (OR 3.7, p = 0.022). However, this was considered to be attributable to the underlying conditions of patients receiving opioids, rather than any interaction with TachoSil.

## Discussion

This prospective, non-interventional European surveillance study, conducted in a real-world clinical setting, suggests that patients undergoing surgery who receive TachoSil are not at increased risk of TEEs, major bleeding or immunological events.

The study cohort was representative of patients treated with TachoSil in clinical practice. The most common type of surgery was hepatic, which is the most documented use of TachoSil and was the most frequently observed type of surgery in a previous surveillance study [11]. Almost all patients (94%) received TachoSil for haemostasis, with the other 6% receiving TachoSil for sealing purposes, such as the prevention of air leakage.

TachoSil did not appear to be associated with an increased risk of TEEs, with only three patients experiencing a TEE that was considered by the reporting physician to be at least possibly related to TachoSil. Possible causal relationships between TachoSil and AEs were not reviewed by the DMC. The overall reported incidence of TEEs in this study (1.5%) is much lower than the expected 10–15%, which was based on a review of the available literature. However, this expected incidence may have been an over-estimate. Meta-analyses of thromboprophylaxis in major surgery suggest that the incidence of TEEs in patients receiving unfractionated or low-molecular-weight heparin is only around 5% [12-14]. Moreover, the incidence of clinically detected TEEs is only 1–2%, given that the majority of TEEs are clinically silent and only detected using a systematic approach. Thus, the reported 1.5% occurrence in this study is consistent with previous observations, given that the majority of patients (79%) received heparins during or after surgery and that no systematic testing for TEEs was done.

Under-reporting of reportable AEs is a concern in large-scale non-interventional studies, since more events may go unnoticed and the recording of events in medical records may be less rigorously implemented compared with a controlled trial setting. In addition, events that are reported may be missed when data are transferred to the study due to inadequate staff training and monitoring and less intensive source data verification. In particular, the follow-up of patients transferred to other hospitals after surgery can be challenging and may lead to under-reporting of AEs. This was a particular concern in this study for those countries where 6-month follow-up could not be done through direct contact with patients, but only through review of medical records.

However, source data verification of 10% of patients did not suggest that a significant number of AEs were missed, with a similar occurrence of events reported for patients with different end digits of their patient number (Chi-squared test, p = 0.8). In addition, incidences of AEs were similar in patients followed-up by personal contact and those who only had their medical records reviewed. These findings suggest that under-reporting was not a significant problem in this study.

The reported 2% incidence of major bleeding is in line with our experience. However, the protocol defined major bleeding as requiring interventional treatment. This definition may have been imprecise, especially given the heterogeneity of the cohort and range of surgical procedures. For instance, it is possible that major bleeding was considered in subjective terms, only being reported if in excess of what was expected. Transfusions were given to 29% of patients; however, transfusions are not only given because of a major bleeding, but may be given because of disease-related anaemia (e.g. in patients with cancer) or as a routine preventative measure for fluid and haemodynamic reposition, despite increasing evidence that this common clinical practice may be associated with increased morbidity and mortality [15].

Peri-operative anti-coagulant prophylaxis was reported in 80% of patients. The lack of anti-coagulant use in the remaining 20% may be largely attributable to under-reporting of therapy. However, it is also possible that a trend towards earlier post-operative mobilisation of patients following surgery, at least in certain countries, may have reduced the use of prophylactic anti-coagulant therapy.

Another potential limitation with the design of this study was the issue of patient consent. All patients had to provide written informed consent, which could be obtained either before or after surgery. However, obtaining consent before surgery would have meant it was necessary to obtain consent from large numbers of patients who did not subsequently receive TachoSil, a considerable burden on the time and resources of participating centres. More importantly, obtaining consent before surgery carried the risk that surgeons may have been encouraged to use TachoSil in situations where they otherwise may not have done so. To avoid this, Ethics Committees in certain countries (Austria, Denmark and Germany) specified that consent must be obtained after surgery, in order to prove that surgeons' were not influenced in their decision to use TachoSil. However, obtaining consent after surgery meant that patients who died during surgery could not be included and AEs in these patients would not be reported, potentially resulting in selection bias and an underestimation of mortality and AEs. Illustrating this dilemma, six local Ethics Committees in Spain specified consent had to be obtained before surgery, while one stated that consent must be obtained post-surgery. In practice, almost all consents were obtained after surgery. However, since almost all reported TEEs occurred in the post-surgical phase, and since only 10% of TEEs and major bleedings had fatal consequences, the order of magnitude of any possible selection bias was likely to have been small.

Occurrence of immunological events was low (<1%), suggesting there is no immunological risk with TachoSil. Hypersensitivity or allergic reactions may occur in rare cases in patients treated with fibrin sealant, especially if the preparation is applied repeatedly. Of particular concern, cross-reaction of antibodies against bovine thrombin and factor V with human factor V has been widely reported after exposure to topical bovine thrombin [16-18]. The risk of immunological reaction to TachoSil is minimal since the fibrinogen and thrombin are of human origin (no bovine material), while the equine collagen patch is free of immunogenic epitopes and is unlikely to induce an immune response [19]. It was impossible to ascertain whether patients had previous exposure to TachoSil. Seven patients reported that they had previously participated or were participating in another clinical study of TachoSil, although it was not possible to confirm whether these patients were actually treated with TachoSil. Two patients received TachoSil on two occasions during this study. None of these patients experienced an immunological event.

Since the active ingredients of TachoSil are derived from human plasma, and the equine collagen fleece is derived from horse tendons, the potential for virus infection has to be considered. Standard measures to prevent infections resulting from the use of medicinal products prepared from human blood or plasma include selection of donors, and screening of individual donations and plasma pools for specific markers of infection. In addition, the manufacture of TachoSil includes several processing steps (e.g. pasteurisation, precipitation and adsorption, polymerase-chain-reaction, sterilisation by gamma irradiation) intended to reduce the risk of viral transmission. No viral infections have been reported as AEs in clinical studies of TachoSil and no proven virus transmission has been detected up to the present since its approval in Europe.

There was no indication of any drug interactions between TachoSil and concomitant medications, as assessed by participating physicians and a post-hoc multiplicity corrected analysis of ORs of number of patients with and without events for each concomitant drug class.

## Conclusion

In conclusion, TachoSil does not appear to be associated with an increased risk of TEEs, major bleeding or immunological events in patients undergoing surgery. In addition, there is no evidence of any interaction between TachoSil and other medications resulting in an increased risk of TEEs or major bleeding. These findings support previous experience that the use of TachoSil in a variety of surgical procedures is safe and well tolerated.

## Competing interests

MB, JF, SB, KG, DM and FM declare they have no competing interests. TT is an employee of Nycomed.

## Authors' contributions

KG, DM and FM comprised the Data Monitoring Committee, which provided input to the study protocol and was responsible for reviewing and evaluating study procedures and data. SB, MB and JF participated in the study and comprised the Publication Committee, which was responsible for preparation and review of the manuscript. TT was responsible for statistical analyses. All authors participated in the review, development and approval of the final manuscript.
